# A Nomogram Based on Clinicopathologic Features and Preoperative Hematology Parameters to Predict Occult Peritoneal Metastasis of Gastric Cancer: A Single-Center Retrospective Study

**DOI:** 10.1155/2020/1418978

**Published:** 2020-12-09

**Authors:** Chao Yang, Yujie Yang, Xiaodong Huang, HuaLi Li, Huangrong Cheng, Shilun Tong, Yongbin Zheng

**Affiliations:** Department of Gastrointestinal Surgery, Renmin Hospital of Wuhan University, Wuhan, 430060 Hu Bei Province, China

## Abstract

**Background:**

In patients with gastric cancer (GC), peritoneal metastasis is an indication of the end stage and often indicates a poor outcome. The diagnosis of peritoneal metastasis, especially occult peritoneal metastasis (OPM), remains a challenge for surgeons. This study was designed to explore the relationship between OPM and clinicopathological characteristics and preoperative hematological parameters in patients with GC and to develop a nomogram to predict the probability of OPM before surgery.

**Methods:**

A total of 672 patients with GC from our center were included, including 583 OPM-negative and 89 OPM-positive patients. These patients were divided into training and validation groups based on when they received treatment. OPM was diagnosed during surgery in patients without any signs of metastasis through imaging examination. Predictive factors were screened by least absolute shrinkage and selection operator logistic regression of all 18 characteristics. The nomogram of OPM was constructed based on these filtered variables. The discriminative and calibration performance of the model were simultaneously evaluated.

**Results:**

A total of six variables, including tumor size, degree of differentiation, depth of invasion, Glasgow prognosis score, and plasma levels of CA125 and fibrinogen, were selected for integration into the final predictive nomogram. The area under curve (AUC) of the nomogram with six factors was 0.906 (95% confidence interval (CI): 0.872-0.941) and 0.889 (95% CI: 0.795-0.984) in the training and validation groups, respectively. Calibration plots of the nomogram in the two sets revealed a good consistency between predicted and actual probabilities. Decision curve analysis showed that the nomogram had a positive net benefit among all threshold probabilities between 0% and 82%. This nomogram was superior to models incorporating only clinicopathologic or hematologic features.

**Conclusion:**

Both clinicopathological and preoperative hematological parameters are significantly associated with OPM. The nomogram constructed with six factors could be used to calculate the probability of OPM and identify the high-risk population in GC. This may be helpful for early detection of OPM in patients with GC.

## 1. Introduction

Gastric carcinoma (GC) is the fifth most common cancer and one of the leading causes of cancer-related death worldwide, especially in East Asia [1]. Peritoneal metastasis, the major style of distant metastasis in patients with GC, is responsible for over half of the mortality from GC [2]. In GC, approximately 10%–30% of peritoneal metastasis can only be discovered during laparoscopy [3, 4]. In patients with GC, occult peritoneal metastasis (OPM) is a special type of peritoneal metastasis that is diagnosed during surgery without any signs of metastasis through imaging examination [5]. OPM includes two conditions, namely, cytology positive (CY1) and macroscopic metastatic lesions (P1) [6]. Hence, OPM could be considered as an early stage of metastasis. Patients with OPM have a more aggressive treatment regimen and better outcome than do those with extensive metastatic lesions [6, 7]. Consequently, accurate and timely preoperative evaluation is crucial for doctors to choose the most appropriate therapies. However, the early detection of metastatic sites, especially for potential peritoneal metastases, remains a challenge for surgeons [8]. Conventional imaging examinations for peritoneal metastasis detection, including computerized tomography (CT) or magnetic resonance imaging (MRI), have insufficient sensitivity and specificity [9–11]. Although diagnostic laparoscopy is a minimally invasive examination with many advantages, clinicians took conservative attitudes on its wide application for peritoneal metastasis detection because of its procedure-related complications [12–14]. Therefore, it is urgent to develop an appropriate noninvasive preoperative detection method as a complementary diagnostic tool for OPM in GC by combining multiple parameters.

Previous studies have indicated that the inflammatory level is closely related to tumor proliferation, invasion, and metastasis in GC [15, 16]. The most commonly used indicators of host inflammation level, including neutrophil-to-lymphocyte ratio (NLR), platelet-to-lymphocyte ratio (PLR), and Glasgow prognosis score (GPS), are considered novel predictors in many cancer types [17–19]. Using simple inflammatory cell counts for predicting cancer progression and for prognostic assessment has gradually received increased attention in GC [20, 21]. Whether NLR, PLR, and GPS can predict OPM in GC has not been assessed. Moreover, correlations between coagulation factors and cancers can date back for over a century. More than 95% of metastatic malignancies have coagulation function abnormalities, and cancer-induced hypercoagulability might accelerate tumor progression and dissemination [22]. Therefore, coagulation data should also be considered in this prediction model. A systematic review suggested that although serum tumor biomarkers cannot detect cancer in its early stage, they are useful for monitoring recurrence and predicting peritoneal/liver metastases [23]. Changes in the levels of tumor biomarkers usually occur 2-3 months before imaging abnormalities can be detected. Therefore, these new findings provide a theoretical basis for OPM detection as early as possible by combining multiple parameters.

In this study, we reviewed patients with GC and OPM in our center and explored factors associated with OPM. The aim of this study was to establish a noninvasive and low-cost prediction model that might be useful for the early detection of OPM.

## 2. Methods

### 2.1. Patients

This is a retrospective study. Patients who underwent laparoscopy from January 2014 to April 2019 in the Department of Gastrointestinal Surgery, Renmin Hospital of Wuhan University, were included. All patients were diagnosed with GC by upper gastrointestinal endoscopy and pathological examination.

The inclusion criteria are as follows: (1) gastric cancer diagnosed by pathology; (2) no evidence indicating tumor invasion of adjacent organs or distant metastasis by preoperative evaluation; and (3) patients were deemed to be suitable for potential curative resection by a multidisciplinary team. The exclusion criteria are as follows: (1) patients had other cancers at the same time; (2) patients received radiotherapy and/or chemotherapy before surgery; (3) patients had any other serious infectious diseases; (4) patients had any blood system diseases and/or had taken medicine that affected coagulation function; and (5) patients had undergone surgery in emergency condition. Included patients were divided into two sets based on the different treatment times.

Patients treated between January 2014 and July 2018 were the training group, and patients treated from August 2018 to April 2019 were the validation group. This study was approved by the Ethics Committee of Renmin Hospital of Wuhan University, and follows the principles of the Declaration of Helsinki. Patient information was anonymized and treated confidentially.

### 2.2. Variables

Patients were assessed before surgery by physical examinations, chest X-ray, abdominal and pelvic contrast-enhanced CT, and blood tests. Venous blood samples were obtained from the patients the morning after admission. Preoperative laboratory data was acquired, including blood cell count, C-reactive protein (CRP), albumin, gastrointestinal tumor biomarkers, and coagulation function tests. Gender and age were also recorded as general characteristics. Tumor-related data included primary tumor location, tumor size, and preoperative clinical staging as determined by CT. Histopathological parameters, including histological types and differentiation degree, were assessed using the preoperative biopsy tissues.

Gastric signet ring cell carcinoma or mucinous adenocarcinoma were defined as nonadenocarcinoma. Clinical T4 was defined as serosa invasion, and clinical T1, T2, and T3 were defined as nonserosa invasion. The GPS ranged from 0 to 2. GPS was a 2 when the CRP level ≥ 10 mg/L and albumin level < 35 mg/L. If only one of CRP or albumin was abnormal, the GPS was 1. If neither CRP nor albumin was abnormal, the GPS was 0.

### 2.3. Surgical Procedure

The laparoscopy procedure was performed as previously described [12]. All laparoscopy procedures were performed by experienced surgeons at the Department of Gastrointestinal Surgery at Renmin Hospital, Wuhan University.

### 2.4. OPM Diagnostic Criteria

OPM was diagnosed when there was no evidence of peritoneal metastasis before surgery, but such metastasis was confirmed during surgery, including CY1 and P1. The weakly diagnostic sensitivity of frozen sections during surgery meant that CY1 and P1 diagnosis depended on exhaustive pathological examination.

### 2.5. Statistical Analysis

Statistical analysis was conducted using the IBM SPSS Statistics software (version 22) and R (version 3.6.0.). The Youden index was used as the optimal cutoff value of each continuous parameter assessed by the receiver operating characteristic (ROC) curve. The chi-square or Fisher tests were used to analyze categorical variables between OPM-positive and OPM-negative groups. Predictive factors were selected by least absolute shrinkage and selection operator (LASSO) regression, and the OPM nomogram prediction models were constructed based on these factors. The area under the curve (AUC) of ROC curves was used to evaluate the prediction model discrimination capability, and the Hosmer-Lemeshow test was used to measure goodness of fit. The calibration capability of the model was evaluated by bootstrap resampling, and the decision curve was used to estimate the clinical net benefit for patients. ROCs were compared using the DeLong test. All *P* values were two-sided and *P* < 0.05 was considered statistically significant.

## 3. Results

### 3.1. Clinical Characteristics and Hematologic Parameters of Patients with GC and OPM

A total of 672 patients with GC who met the inclusion criteria were included in this study. This included 526 in the training group and 146 in the validation group. In the training group, 73 cases were OPM positive and 453 cases were OPM negative. In the validation group, 130 cases were OPM negative and 16 cases were OPM positive. The OPM-positive rate in the training and validation groups were 13.9% and 10.9%, respectively, and did not significantly differ (*P* = 0.157).

Univariate analysis results revealed that clinicopathological characteristics, including tumor size and depth of invasion, significantly correlated with OPM (*P* < 0.05; Table 1). Moreover, OPM was significantly associated with many hematological parameters (Table 2).

### 3.2. Feature Selection

**Figure 1:**
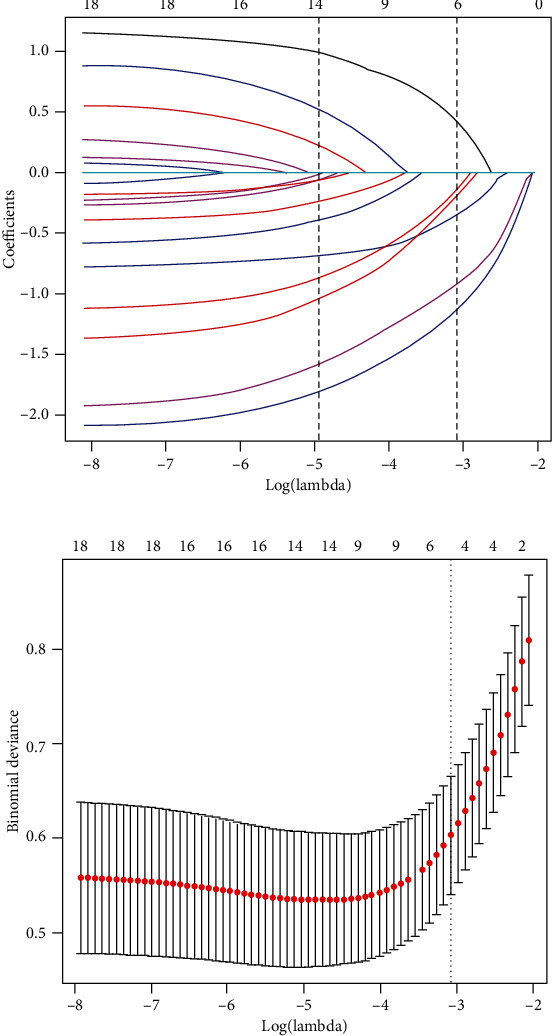
Predictive features were selected using the LASSO binary logistic regression model. (a) LASSO coefficient of the 18 OPM-associated predictors. (b) Feature selection using the LASSO model and 10-fold cross-validation via minimum criteria. The model had excellent performance and the least number of independent variables when lambda was -3.082.

All 18 potential predictors were incorporated into the LASSO logistic regression model for the 526 patients in the training set (Figure 1). Then, six predictors with nonzero coefficients were selected when lambda = −3.082 (lambda · 1se). The six features were tumor size, degree of differentiation, depth of invasion, GPS, and plasma levels of CA125 and fibrinogen.

### 3.3. Construction of the Nomogram for Predicting OPM in Patients with GC

**Figure 2:**
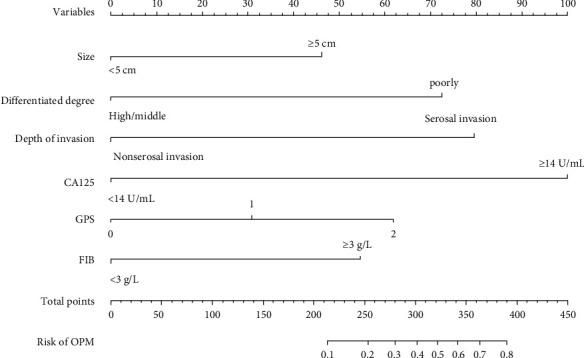
Nomogram for the probability of OPM based on the six predictors: tumor size, degree of differentiation, depth of invasion, GPS, and plasma levels of CA125 and fibrinogen. OPM: occult peritoneal metastasis; CA125: carbohydrate antigen 125; GPS: Glasgow prognosis score; FIB: fibrinogen.

Those six predictors were added into the final nomogram (named Model A, Figure 2). Each factor in the nomogram was assigned a weighted score. For each patient, the total points were associated with the probability of OPM. Considering that some included parameters mentioned above may not be assessed in other centers, two models with only clinicopathological or hematological features (Model B and Model C, respectively) were also built and evaluated simultaneously.

### 3.4. Nomogram Performance in the Training and Validation Groups

The coefficients of variables in three models are shown in Table 3. The combined model (Model A) had lower Akaike information criterion (AIC) than did Model B and Model C, indicating the best fit. The categorical net reclassification improvement (NRI) revealed that Model A had better predictive value than did Model B and Model C in the training cohort (Model A vs. Model B: 0.160 (95% CI: 0.067-0.253), *P* < 0.001; Model A vs. Model C: 0.177 (95% CI: 0.060-0.293), *P* = 0.003). In the validation group, the categorical NRI revealed that Model A had better predictive value than did Model C, but was not markedly better than Model B (Model A vs. Model C: 0.171 (95% CI: 0.001-0.341), *P* = 0.049; Model A vs. Model B: 0.093 (95% CI: -0.128 to -0.315), *P* = 0.409).

**Figure 3:**
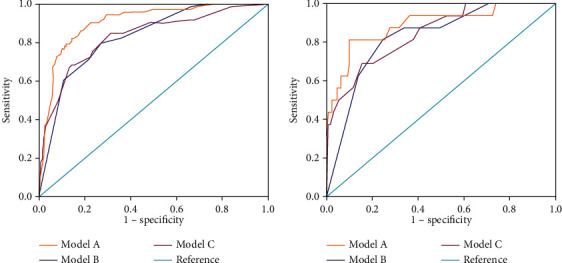
The discriminative abilities of Models A, B, and C were evaluated using the AUC of ROCs. Model A (including clinicopathological and preoperative hematological parameters) had the best performance both in the training (a) and validation (b) cohorts (DeLong's test, *P* < 0.05).

The predictive accuracy of the nomogram was visually displayed by ROC curves (Figure 3). In the training group, Model A yielded the highest AUC (0.906 (95% CI: 0.872-0.941), indicating that this model has good discrimination and reliable ability as a predictive tool for OPM. The AUCs for Models B and C were 0.828 (95% CI: 0.781-0.875) and 0.832 (95% CI: 0.778-0.886), respectively. The DeLong test revealed that the AUCs significantly differed between Model A and the other two models (both *P* < 0.001 for Model A vs. Model B or Model C). In the validation group, Model A had the highest AUC (0.889 (95% CI: 0.795–0.984), while Models B and C had AUCs of 0.827 (95% CI: 0.730–0.925) and 0.845 (95% CI: 0.748–0.943). The DeLong test revealed statistically significant differences in the AUCs of Model A and Model B (*P* = 0.046), but not between Model A and Model C (*P* = 0.209).

**Figure 4:**
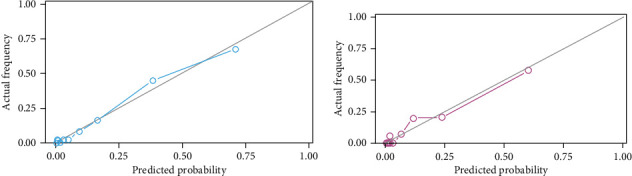
Nomogram calibration plots. (a) Calibration plot of the nomogram in the training set showed that the predicted and actual probabilities were similar. (b) In the validation cohort, the nomogram calibration plot revealed the consistency of the model (both Hosmer-Lemeshow's test, *P* > 0.05).

The calibration plot suggested a good concordance between the predicted and observed values for Model A in both the training and validation sets (Figure 4). This was supported by the Hosmer-Lemeshow test results (*χ*^2^ = 5.180, *P* = 0.819; *χ*^2^ = 3.359, *P* = 0.948 for training and validation sets, respectively).

### 3.5. Clinical Use

**Figure 5:**
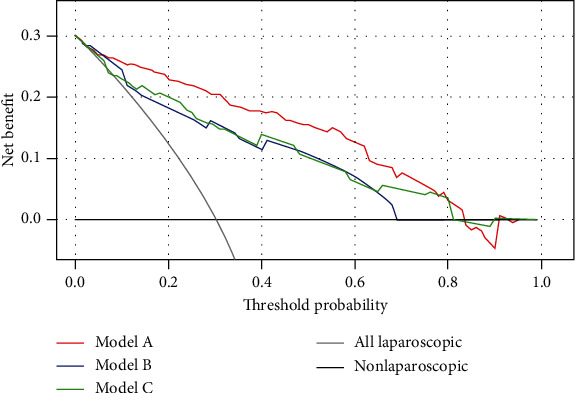
The net benefit curves for the three nomogram models in the training group. Model A had a positive net benefit when thresholds were between 0 and 82%.

Decision curve analysis was based on the three nomogram models in the training set (Figure 5). Model A had the maximum clinical net benefit. The published literature indicated that patients would need to receive specialized interventions (usually laparoscopy scheme) when the preoperative predicted probabilities of peritoneal metastasis are greater than 30% [12]. Compared with patients who underwent all laparoscopic or nonlaparoscopic interventions, the patient could obtain an additional clinical net benefit of approximately 20% by the use of Model A for clinical decision-making.

## 4. Discussion

The average survival time of GC patients with peritoneal dissemination is less than six months, and these patients cannot achieve benefit from conventional therapy [24]. Typical signs, such as massive ascites or abdominal masses, are commonly used to diagnose peritoneal metastasis in patients with GC during clinical practice. However, these patients are often in the terminal stage of the disease and have poor clinical prognoses [7]. If peritoneal metastasis was accurately diagnosed in an early stage, the survival time of these patients would be improved. Unfortunately, the lack of specific and sensitive radiological examination approaches makes the accurate preoperative diagnosis of peritoneal metastasis of GC difficult [25–27]. In the absence of a single effective evaluation, there is a need for the construction of a model combining low cost and easily available laboratory parameters with radiological characteristics. The nomogram constructed in this study might provide a simple tool to predict the probability of OPM. This could be very useful for the clinical screening of high-risk populations for OPM and deciding whether or not to perform diagnostic laparoscopy.

As a simple class of blood cell parameters, NLR and PLR could be easily determined from routine laboratory work and used to accurately reflect the level of systemic inflammation [28]. Preoperative NLR or PLR are strongly independent predictors of extensive peritoneal metastasis in advanced gastric cancer [29–31]. Although NLR and PLR were significantly correlated with OPM in this study, they were not included in the final nomogram as independent predictors of OPM. One reasonable explanation for this is that OPM might be considered the initial stage of extensive peritoneal metastasis lesions.

GPS is also an easily accessible and reliable marker that indirectly reflects the host inflammatory level using serum CRP and albumin. Patients with peritoneal metastases generally have higher CRP levels [32]. Such patients usually also have cachexia because of inadequate nutritional intake, frequent bleeding, or massive ascites, which aggravate hypoproteinemia. All of these may cause the GPS value to rise. Moreover, GPS was also closely correlated with the degree of peritoneal metastasis (*P* = 0.001) and volume of ascites (*P* < 0.001) [33]. This study also confirmed that GPS plays a key role in predicting OPM (OR = 3.814).

The tiny isolated lesions in the peritoneal cavity mean that tumor biomarkers might be better tools than examination using images [34]. Preoperative plasma CA125 is one of the most reliable clinical markers in the diagnosis and prognosis evaluation of patients with peritoneal dissemination [35]. Emoto et al. reported that the sensitivity of CA125 for peritoneal metastasis at initial diagnosis was 45%, and the median survival time of patients with lower CA125 levels was significantly longer than that of patients with higher levels [34]. In this study, higher serum CA125 was significantly correlated with OPM in patients with GC, and the odds ratio was significant at 5.355. Immunohistochemical analysis showed that CA125 expression on the surface of gastrointestinal malignant tumor cells was not common [36]. CA125, a large-molecule type-I transmembrane glycoprotein, only exists on the surface of some mesothelial cells which are the main cellular components of the peritoneum. This means that increased plasma CA125 level is not solely from the growth of the tumor focus, but reflective of the degree of peritoneal mesothelial cellular injury triggered by carcinomas [37].

The “seed and soil” hypothesis put forward by Paget [38] has been widely accepted as the fundamental theory of peritoneal dissemination in GC. In this theory, peritoneal-free cancer cells, which are detached from the primary tumor, are compared to “seeds” and the suitable environment of the tumor cancers are compared to “soil.” The frequency of peritoneal metastasis should increase significantly because more free cancer cells are exfoliated from the primary tumor when the tumor cells penetrate the gastric serosa. Li et al. performed a prospective study of diagnostic laparoscopy for 249 cM0 and suggested that the depth of invasion was an independent risk factor for intraperitoneal metastasis [39]. This study showed that 83.8% of OPM were cT4, indicating that tumor cells directly enter the abdominal cavity and adhere to the peritoneum to develop extensive peritoneal metastasis. Interestingly, Yoshida and Huang et al. [40, 41] reported a small number of early non-serosa-invasive cases with peritoneal dissemination and provided a novel possible mechanism through which primary tumor cells could be indirectly shed into the peritoneal cavity via lymphatic networks on the peritoneum.

As a rising technology in recent years, radiomics has the advantages of being noninvasive and producing large volumes of information. Recently, Dong et al. developed an OPM prediction model based on radiomics features, including three predictors (RS1, RS2, and Lauren's type), and the model showed an excellent ability to predict OPM (AUC = 0.958) [7]. With the continuous improvement of detection equipment and algorithm technology, a promising diagnostic method for OPM could arise from combining radiologic characteristics and other clinicopathological parameters [42, 43].

## 5. Conclusion

Both clinicopathological and preoperative hematological parameters are significantly associated with OPM. The nomogram constructed in this study could effectively predict the incidence of OPM. It is helpful to identity high-risk patients with OPM and provide a guide for optimal treatment strategies and avoid unnecessary operative treatments. However, there are some limitations in the study. Due to the small size of the samples in the validation set, the baseline characteristics were not well-matched between the training and validation sets. Another limitation to this study was that it was a single-center study with retrospective data collection. Therefore, the influence of selection bias should be considered when using this prediction nomogram in other centers.

## Figures and Tables

**Table 1:** Demographic and clinicopathological characteristics of patients.

Various	Training group			Validation group		
	OPM (+)	OPM (-)	*P* value	OPM (+)	OPM (-)	*P* value
Gender, no. (%)			0.555			0.135
Female	21 (28.8)	146 (32.2)		9 (56.3)	48 (36.9)	
Male	52 (71.2)	307 (67.8)		7 (43.8)	82 (63.1)	
Age, no. (%)			0.900			0.512
<60	36 (49.3)	227 (50.1)		7 (43.8)	61 (46.9)	
≥60	37 (50.7)	226 (49.9)		9 (56.3)	69 (53.1)	
Location, no. (%)			0.209			0.430
Lower third	36 (49.3)	259 (57.2)		11 (68.8)	74 (56.9)	
Upper/middle third	37 (50.7)	194 (42.8)		5 (31.3)	56 (43.1)	
Size, no. (%)			*<0.001*			*0.003*
<5 cm	20 (27.4)	311 (68.7)		5 (31.3)	93 (71.5)	
≥5 cm	53 (72.6)	142 (31.3)		11 (68.8)	37 (28.5)	
Pathological type, no. (%)			0.971			0.622
Adenocarcinoma	62 (84.9)	384 (84.8)		14 (87.5)	120 (92.3)	
Nonadenocarcinoma	11 (15.1)	69 (15.2)		2 (12.5)	10 (7.7)	
Differentiated degree, no. (%)			*<0.001*			0.102
High/middle	9 (12.3)	203 (44.8)		5 (31.3)	67 (51.5)	
Poorly	64 (87.7)	250 (55.2)		11 (68.8)	63 (48.5)	
Depth of invasion, no. (%)			*<0.001*			*<0.001*
Nonserosal invasion	13 (17.8)	305 (67.3)		3 (18.8)	85 (65.4)	
Serosal invasion	60 (82.2)	148 (32.7)		13 (81.3)	45 (34.6)	

OPM (+): with occult peritoneal metastasis. OPM (-): without occult peritoneal metastasis. Gastric signet ring cell carcinoma or mucinous adenocarcinoma was defined as nonadenocarcinoma. cT4 was defined as serosal invasion, and cT1, cT2, and cT3 were defined as nonserosal invasion.

**Table 2:** Univariate analysis of preoperative hematology parameters in groups with or without occult peritoneal metastasis.

Various	Training group		*P* value	Validation group		*P* value
	OPM (+)	OPM (-)		OPM (+)	OPM (-)	
NLR			*<0.001*			0.797
<2	20 (27.4)	224 (49.4)		9 (11.7)	68 (52.3)	
≥2	53 (72.6)	229 (50.6)		7 (10.1)	62 (47.7)	
PLR			*<0.001*			0.585
<165	33 (45.2)	302 (66.7)		9 (56.3)	84 (64.6)	
≥165	40 (54.8)	151 (33.3)		7 (13.2)	46 (35.4)	
CEA			*0.049*			0.530
<2.5 ng/mL	49 (67.1)	352 (77.7)		11 (68.8)	101 (77.7)	
≥2.5 ng/mL	24 (32.9)	101 (22.3)		5 (31.3)	29 (22.3)	
CA199			*0.001*			0.521
<30 U/mL	46 (63.0)	364 (80.4)		14 (87.5)	109 (83.8)	
≥30 U/mL	27 (37.0)	89 (19.6)		2 (12.5)	21 (16.2)	
CA125			*<0.001*			*0.003*
<14 U/mL	16 (21.9)	329 (72.6)		3 (18.8)	77 (59.2)	
≥14 U/mL	57 (78.1)	124 (27.4)		13 (81.3)	53 (40.8)	
GPS			*<0.001*			*0.001*
0	20 (27.4)	196 (43.3)		3 (18.8)	44 (33.8)	
1	34 (46.6)	230 (50.8)		5 (31.3)	79 (60.8)	
2	19 (26.0)	27 (6.0)		8 (53.3)	7 (5.4)	
PT			0.811			0.670
<12 sec	54 (74.0)	341 (75.3)		14 (87.5)	117 (90.0)	
≥12 sec	19 (26.9)	112 (24.7)		2 (12.5)	13 (10.0)	
APTT			0.467			0.566
<28 sec	44 (60.3)	293 (64.7)		10 (62.5)	92 (70.8)	
≥28 sec	29 (39.7)	160 (35.3)		6 (37.5)	38 (29.2)	
TT			0.219			0.786
<18 sec	47 (64.4)	257 (56.7)		11 (68.8)	82 (63.1)	
≥18 sec	26 (35.6)	196 (43.3)		5 (31.3)	48 (36.9)	
FIB			*<0.001*			*0.005*
<3 g/L	26 (35.6)	278 (61.4)		4 (25.0)	83 (63.8)	
≥3 g/L	47 (64.6)	175 (38.6)		12 (75.0)	47 (36.2)	
D-dimer			0.078			0.054
<0.5 mg/L	36 (49.3)	273 (60.3)		6 (37.5)	84 (64.6)	
≥0.5 mg/L	37 (50.7)	180 (39.7)		10 (62.5)	46 (35.4)	

OPM (+): with occult peritoneal metastasis; OPM (-): without occult peritoneal metastasis; NLR: neutrophil-to-lymphocyte ratio; PLR: platelet-to-lymphocyte ration; CEA: carcinoembryonic antigen; CA199: carbohydrate antigen 19-9; CA125: carbohydrate antigen 125; GPS: Glasgow prognosis score; PT: prothrombin time; APTT: activated partial thromboplastin time; TT: thrombin time; FIB: fibrinogen.

**Table 3:** Coefficients of six variables in three nomogram models.

Various	Model A			Model B			Model C		
	*β*	OR (95% CI)	*P*	*β*	OR (95% CI)	*P*	*β*	OR (95% CI)	*P*
Size (≥5 cm)	0.839	2.313 (1.130-4.737)	0.022	1.194	3.300 (1.802-6.043)	<0.001	—	—	—
Differentiated degree (poorly)	1.420	4.137 (1.743-9.817)	0.001	1.436	4.202 (1.963-8.997)	<0.001	—	—	—
Serosal invasion	1.669	5.304 (2.509-11.214)	<0.001	1.781	5.938 (3.028-11.645)	<0.001	—	—	—
CA125 (≥14 U/mL)	1.950	7.026 (3.541-13.937)	<0.001	—	—	—	2.171	8.767 (4.627-16.613)	<0.001
CA199 (≥30 U/mL)	—	—	—	—	—	—	0.741	2.098 (1.127-3.904)	0.019
GPS (2)	1.339	3.814 (1.372-10.599)	0.010	—	—	—	1.517	4.558 (1.829-11.355)	0.001
FIB (≥3 g/L)	1.012	2.752 (1.423-5.322)	0.003	—	—	—	1.062	2.893 (1.567-5.343)	0.001
AIC	277.32			331.76			335.41		

Model A: includes all six predictors; Model B: includes clinicopathological features only; Model C: includes hematologic characteristics only; CA199: carbohydrate antigen 19-9; CA125: carbohydrate antigen 125; GPS: Glasgow prognosis score; FIB: fibrinogen. AIC: Akaike information criterion.

## Data Availability

The data used to support the findings of this study are available from the corresponding author upon reasonable request.
